# MASTL promotes cyclin B1 destruction by enforcing Cdc20-independent binding of cyclin B1 to the APC/C

**DOI:** 10.1242/bio.201410793

**Published:** 2015-03-06

**Authors:** Erik Voets, Rob Wolthuis

**Affiliations:** 1 Division of Cell Biology I (B5) and Division of Molecular Carcinogenesis (B7), The Netherlands Cancer Institute (NKI-AvL), Plesmanlaan 121, 1066 CX Amsterdam, The Netherlands; 2 Section of Oncogenetics, Department of Clinical Genetics and CCA/V-ICI Research Program Oncogenesis, VUmc Medical Faculty, van de Boechorststraat 7, 1081 BT Amsterdam, The Netherlands

**Keywords:** Greatwall, MASTL, Cdk1, Cyclin B1, PP2A, Separase, APC/C^Cdc20^

## Abstract

When cells enter mitosis, the anaphase-promoting complex/cyclosome (APC/C) is activated by phosphorylation and binding of Cdc20. The RXXL destruction box (D-box) of cyclin B1 only binds Cdc20 after release of the spindle checkpoint in metaphase, initiating cyclin B1 ubiquitination upon chromosome bi-orientation. However, we found that cyclin B1, through Cdk1 and Cks, is targeted to the phosphorylated APC/C^Cdc20^ at the start of prometaphase, when the spindle checkpoint is still active. Here, we show that MASTL is essential for cyclin B1 recruitment to the mitotic APC/C and that this occurs entirely independently of Cdc20. Importantly, MASTL-directed binding of cyclin B1 to spindle checkpoint-inhibited APC/C^Cdc20^ critically supports efficient cyclin B1 destruction after checkpoint release. A high incidence of anaphase bridges observed in response to *MASTL* RNAi may result from cyclin B1 remaining after securin destruction, which is insufficient to keep *MASTL*-depleted cells in mitosis but delays the activation of separase.

## INTRODUCTION

Mitosis is regulated by a complex network of kinases and their counteracting phosphatases. Maturation-promoting factor (MPF), better known as cyclin B-Cdk1, triggers mitotic entry by phosphorylating an extensive set of substrates (Errico et al., 2010). These phosphorylations promote DNA condensation, initiate nuclear envelope breakdown (NEB) (Kutay and Hetzer, 2008), and drive spindle assembly. The effects of cyclin B-Cdk1 are enforced by other mitotic kinases, such as Polo-like kinase 1 (Plk1) and Aurora A, that also contribute to the activation of cyclin B-Cdk1 (Macůrek et al., 2008; Seki et al., 2008). The combined action of these kinases directs early mitosis, but subsequent reversal of many of their effector phosphorylations is a prerequisite for mitotic exit and cytokinesis. In higher eukaryotes, the main phosphatase required for Cdk1 substrate dephosphorylation is probably protein phosphatase 2A (PP2A), in complex with its B-type regulatory subunits known as B55 (Manchado et al., 2010; Mochida et al., 2009; Mayer-Jaekel et al., 1994; Schmitz et al., 2010).

Whereas PP2A-B55 appears to be highly active in interphase, it is inhibited during mitosis. PP2A-B55 becomes inactive around the time when cyclin B-Cdk1 is switched on. A kinase called Greatwall (Gwl), also known as microtubule-associated serine/threonine kinase-like (MASTL) in mammals, directs the repression of PP2A in mitosis (Burgess et al., 2010; Castilho et al., 2009; Vigneron et al., 2009; Voets and Wolthuis, 2010; Zhao et al., 2008). Recent work shows that Gwl is activated directly by cyclin B-Cdk1, and possibly also by Plk1, during prophase (Álvarez-Fernández et al., 2013; Wang et al., 2013). Once active, Gwl on its turn supports the further phosphorylation of cyclin B-Cdk1 substrates by restraining PP2A. Work from two independent groups showed that PP2A inhibition by Gwl requires the presence of α-Endosulfine (Ensa) and cAMP-regulated phosphoprotein 19 (Arpp19) (Gharbi-Ayachi et al., 2010; Mochida et al., 2010). These two small homologous proteins are substrates of Gwl that, upon phosphorylation, bind and inhibit PP2A-B55 (Bontron et al., 2013; Juanes et al., 2013; Kim et al., 2012; Rangone et al., 2011; Talarek et al., 2010; Wang et al., 2011). However, evidence supporting the effects of the Endosulfines in mammalian cells remains scarce.

Genetic ablation of *MASTL* in mice results in early embryonic lethality, indicating that Gwl is indispensable for cell division or development (Álvarez-Fernández et al., 2013). Unlike Cdk1, the presence of *GWL* is not strictly essential for entry into mitosis in cultured cells (Álvarez-Fernández et al., 2013; Archambault et al., 2007). Most deficiencies ascribed to *GWL* ablation are mitotic, including defective chromosome condensation, abnormal spindle assembly, and chromosome segregation errors (Archambault et al., 2007; Bettencourt-Dias et al., 2004; Burgess et al., 2010; Voets and Wolthuis, 2010; Yu et al., 2004). Generally, these defects can be restored by partially suppressing PP2A-B55 (Burgess et al., 2010; Rangone et al., 2011), supporting the model that Gwl's main function is to inhibit the activity of this Cdk1-counteracting phosphatase.

PP2A gains activity again when Cdk1 is inactivated during metaphase, which requires recognition of cyclin B1 by Cdc20 and the anaphase-promoting complex/cyclosome (APC/C) (Pines, 2006; Yu, 2007). Interestingly, one of the defects observed after depletion of *MASTL* in human cells is the incomplete degradation of cyclin B1 during mitotic exit (Voets and Wolthuis, 2010).

Here, we investigated how MASTL influences APC/C^Cdc20^. We find that cells can enter mitosis after *MASTL* depletion, but mitotic phospho-serine and phospho-threonine levels are reduced approximately two-fold. When these cells exit mitosis, the APC/C^Cdc20^ substrates geminin and securin are effectively degraded, albeit with some delay. However, approximately 40% of cyclin B1 remains present for at least three hours after mitosis. We show that MASTL particularly supports the efficiency of cyclin B1 destruction because it enforces the Cdc20-independent binding of cyclin B1 to the mitotic APC/C. *MASTL*-depleted cells that exit mitosis also display a high frequency of DNA bridges. Under these conditions, separase may be activated too late, because cyclin B1 molecules remain present in anaphase. Impaired separase activation in combination with ongoing cytokinesis can lead to incomplete sister chromatid separation. We conclude therefore that MASTL is critical to ensure the coordinated degradation of cyclin B1 and securin, which is essential for successful anaphase and maintenance of genomic integrity.

## MATERIALS AND METHODS

### Cell culture and synchronisation

HeLa-ECO (expressing the ecotropic receptor), Phoenix-ECO (expressing gag-pol and an ecotropic envelope protein), and Cdc20(Δ/Δ); RERT(+/Cre) MEFs were cultured in DMEM (Gibco) containing 8% heat-inactivated FBS (Hyclone) supplemented with 100 U/ml penicillin and 100 µg/ml streptomycin at 37°C in 5% CO_2_. 24 or 48 hours before synchronisation, transfection or treatment, cells were seeded on either 6-well Costar plates or 9 cm Falcon dishes. For enrichment of cells in mitosis, cells were treated for 16–24 hours with thymidine (T1895, 2.5 mM final concentration [Sigma-Aldrich]), released into fresh medium and subsequently treated with nocodazole (830 nM final concentration [Sigma-Aldrich]) or taxol (500 nM final concentration [Sigma-Aldrich]) for 16 hours. Alternatively, the Cdc20(Δ/Δ); RERT(+/Cre) MEFs were treated for 9 hours with thymidine due to their high proliferation rate, released, and refreshed with medium containing nocodazole or 4-hydroxytamoxifen (4-OHT) (H7904, 1 µM final concentration [Sigma-Aldrich]. Mitotic cells were collected by mitotic shake-off. For G1 phase synchronisations, mitotic cells were refreshed with medium containing RO-3306 (#217699, 10 µM final concentration [Calbiochem]), or when indicated with AZD1152 (1 µM final concentration [AstraZeneca Pharmaceuticals]). G2 phase cells were collected 9 hours after release from a thymidine block, after washing away any early mitotic cells.

Other drugs in this study are used as indicated: proteasome inhibitor MG132 (#13697, 5 µM final concentration [Cayman Chemicals]); the catalytic Topo2α and Topo2β inhibitor ICRF-193 (I4659, 5 µM final concentration [Sigma-Aldrich]).

### RNAi, transfections and infections

The siRNAs to target cyclin A2 (*CCNA2*), cyclin B1 (*CCNB1*), Cdk1 (*CDC2*), *CDC20*, *CKS1B* and *CKS2* (combined as pool of si*CKS1+2*), separase (*ESPL1*), *CDH1*, *MASTL*, *PLK1*, PP2A-Cα (*PPP2CA*), shugoshin (*SGOL1*), and Topo2α (*TOP2A*) were purchased from Thermo Fisher Scientific as set of four individual ON-TARGET-plus oligos (see supplementary material Table S1 for sequences).

Transfection of siRNA pools were performed using Lipofectamine 2000 (Invitrogen) according to the manufacturer's instructions. Cells seeded into 6-well plates or 9-cm dishes were transfected twice in most experiments (except for the knockdown of *PLK1* and *SGOL1*), with a 24 hour time-interval, using 40 nM siRNA to obtain efficient depletion.

To create stable cell lines, Phoenix-ECO cells were transfected in 6-well plates with 4 µg of pRetroSuper.puro targeting either *GFP* (5′-GCTGACCCTGAAGTTCATC-3′) or *PPP2CA* (5′-GGATAGCAGCAAACAATCA-3′), using the standard calcium phosphate precipitation method. Viral supernatant was collected three times, cleared through a 0.45-µm filter (EMD Millipore), and used to infect HeLa-ECO cells in presence of 5 µg/ml polybrene. Transduced cells were selected on puromycin (2.0 µg/ml) for 3 days, and resistant cells were subcultured to validate successful knockdown on the protein level and used for further experiments.

### Antibodies

The antibodies against the following proteins were used: ANA-Centromere CREST AutoAb Human (Fitzgerald 90C-CS1058), goat anti-Actin (Santa Cruz sc-1616), mouse anti-α-Tubulin (Sigma T5168), mouse anti-APC3 (BD Transduction #610455), mouse anti-APC4 (gift of Jonathon Pines), goat anti-APC4 (Santa Cruz sc-21414), rabbit anti-APC8 (BioLegend 611402), rabbit anti-APC10 (BioLegend 611502), rabbit anti-Aurora A (Cell Signaling #3092), mouse anti-BubR1 (Chemicon MAB3612), mouse anti-Cdc20 (Santa Cruz sc-13162), rabbit anti-Cdc20 (Santa Cruz sc-8358), mouse anti-Cdh1 (Neomarkers #MS-1116-P1), mouse anti-Cdk1 (BD Transduction 610038), rabbit anti-Cdk1 phospho-Tyr15 (Cell Signaling #9111s), rabbit anti-cyclin A2 (Santa Cruz sc-751), mouse anti-cyclin A2 (Neomarkers #MS-1061-S1), mouse anti-cyclin B1 (Santa Cruz sc-245), rabbit anti-cyclin B1 (Santa Cruz sc-752), mouse anti-Emi1 (Zymed 37-6600), rabbit anti-geminin (Santa Cruz sc-13015), mouse anti-GFP (Santa Cruz sc-9996), rabbit anti-GFP (2C, home-made), rabbit anti-Histone H3 phospho-Ser10 (Millipore 06-570), mouse anti-Mad2 (MBL K0167-3), rabbit anti-Mad2 (Bethyl Laboratories A300-300A), rabbit anti-MASTL (Bethyl Laboratories A302-190A), rabbit anti-Nek2 (Santa Cruz sc-33167), rabbit anti-phosho-Threonine (Cell Signaling #9381), rabbit anti-phosho-Serine CDKs substrate (Cell Signaling #2324S), mouse anti-Plk1 (Santa Cruz sc-17783), rabbit anti-PP2A-A (Cell Signaling #2039), mouse anti-PP2A-Cα (Millipore 05-421), rabbit anti-securin (Zymed 34-1500), mouse anti-securin (Abcam ab3305), mouse anti-separase (Abcam ab16170), mouse anti-shugoshin (Novus Biologicals H00151648-B01), and rabbit anti-Topo2α (Bethyl Laboratories A300-054A). Secondary peroxidase-conjugated antibodies were obtained from DAKO and ALEXA fluorescently-labelled secondary antibodies were purchased from Molecular Probes.

### Western blotting and immunoprecipitations

Cells were lysed in ELB+ (150 mM NaCl, 50 mM HEPES (pH 7.5), 5 mM EDTA, 0.3% NP-40, 10 mM β-glycerophosphate, 6% glycerol, 5 mM NaF, 1 mM Na_3_VO_4_ and Roche protease inhibitor cocktail). Lysates were cleared by centrifugation (13,000 ***g***, 10 min at 4°C). Protein levels were equalized by using Bradford analysis. For immunoprecipitations, 20 µl of protein G Sepharose (Amersham Biosciences) were blocked for 24 hours in 1% BSA/PBS containing 0.1% Tween (PBS-T), washed with ELB+, and precoupled with 2 µg antibodies for 16 hours. Precoupled beads were washed with ELB+, incubated for 4 hours in presence of the protein lysates at 4°C, and washed three times with 1.0 ml of ice-cold ELB+. All remaining buffer was then removed and beads were resuspended in 50 µl sample buffer; 25 µl was separated on SDS-PAGE and blotted on nitrocellulose (0.4 µm pore). Membranes were blocked with 5% ELK in PBS containing 0.1% Tween. Development of blots was performed using the Chemidoc Imaging System (Bio-Rad Laboratories) and quantification was done with the Image Lab (Bio-Rad Laboratories) software.

### Immunofluorescence

Cells were grown on glass coverslips and fixed with 3.7% formaldehyde in PBS (containing Ca^2+^ and Mg^2+^) for 10 min followed by permeabilisation for 10 min with 0.1% Triton X-100 in PBS (Ca^2+^ and Mg^2+^). Cells were blocked in PBS (Ca^2+^ and Mg^2+^) containing 10% FBS and labelled with primary antibodies as indicated. Cells were washed 3 times followed by labelling with Alexa Fluor secondary antibodies. DNA staining was performed with 4′,6-Diamidino-2-phenylindole (DAPI [Molecular Probes]) after which the coverslips were mounted in Vectashield solution. Z-stacks with 0.2 µm spacing were acquired using either a Leica AOBS confocal microscope or a DeltaVision Elite system (Applied Precision). Maximum intensity projection of the z-levels were analysed with ImageJ (National Institute of Health) and processed using Photoshop and Illustrator software (Adobe).

### Chromosome spreads

HeLa cells transfected with siRNAs as indicated were synchronised in mitosis by a thymidine block for 16 hours, released into fresh medium and subsequently treated with 830 nM nocodazole for 5 hours. Cells were processed for chromosome spreads 48 hours after transfection (or 24 hours in case of *SGOL1* RNAi). Adherent cells and floating cells were harvested, centrifuged for 5 minutes at 400 g, and supernatant was discarded. Subsequently, cells were resuspended in pre-warmed (at 37°C) 0.075 M KCl, while shaking constantly. Cells were incubated at 37°C for 10 minutes, a small volume of methanol/acetic acid (in a ratio of 3:1) was added dropwise, and cells were centrifuged for 5 minutes at 400 ***g***. The supernatant was discarded and the cell pellet was resuspended in a leftover of the supernatant. For fixation, 1 ml of the methanol/acetic acid solution was added while shaking and this suspension was incubated for 20 minutes at room temperature. Thereafter, the cells were centrifuged for 5 minutes at 400 ***g***. This fixation procedure was repeated 2 times. Lastly, the cell pellet was resuspended in methanol/acetic acid and the suspension was dropped from±30 cm height onto clean microscope slides. The slides were air-dried, covered with DABCO/DAPI solution to stain the DNA, and protected by a glass coverslip.

## RESULTS

### MASTL-directed PP2A inhibition doubles Cdk1 substrate phosphorylation in mitosis

**Fig. 1.**
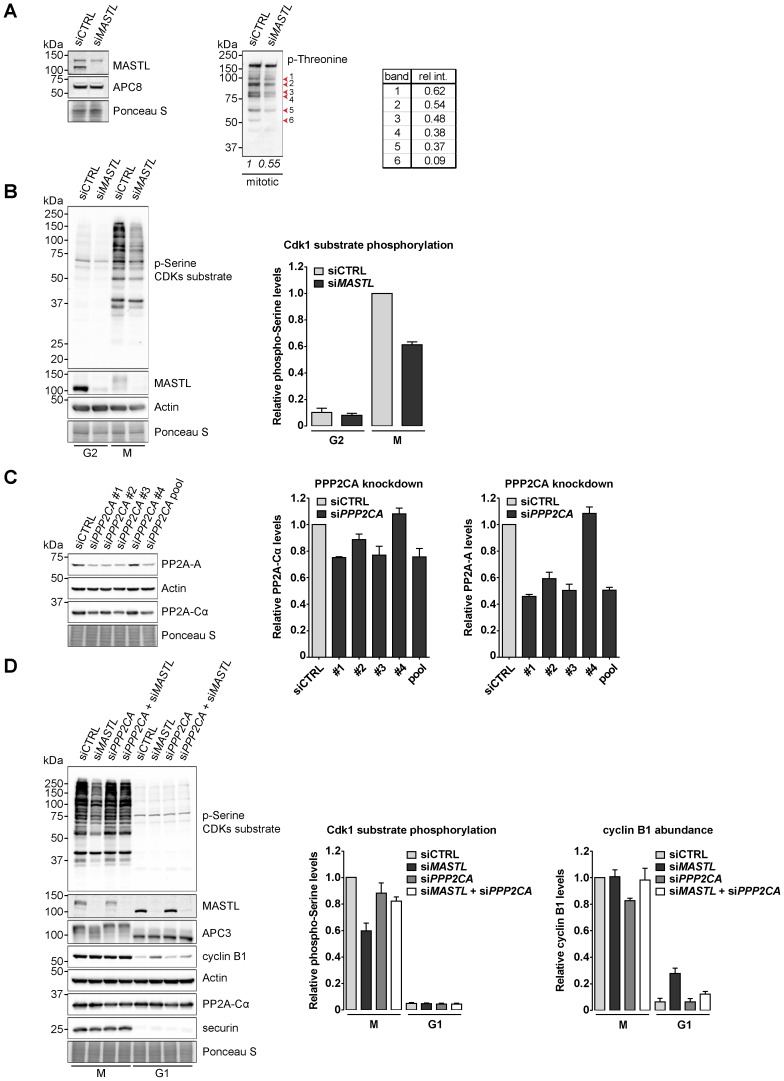
MASTL counteracts the mitotic phosphatase PP2A. (A) Depletion of *MASTL* reduces mitotic phosphorylation events. HeLa cells transfected with a control RNAi (siCTRL) or with a siRNA pool targeting *MASTL* (si*MASTL*) were either asynchronously grown or synchronised in mitosis by a thymidine block and subsequent nocodazole treatment. Cells arrested in mitosis were collected by mitotic shake-off. Extracts were processed for western blot analysis and blotted for the proteins as indicated. A quantification of the phosphorylated threonine signal (p-Threonine) demonstrates that *MASTL* depletion lowers the abundance of mitotic phosphorylations. The intensity of the p-Threonine signal was quantified for the entire lane, as well as for individual bands on the gel. (B) MASTL contributes to efficient Cdk1 substrate phosphorylation. HeLa cells transfected with indicated siRNAs were synchronised in G2 and M phase by thymidine synchronisation and subsequently blocked in mitosis by nocodazole treatment. Mitotic cells were collected by mitotic shake-off. Extracts were processed for western blot analysis and blotted for the proteins as indicated. The intensity of the phosphorylated serine Cdks substrate antibody signal (p-Serine CDKs substrate) was quantified for each individual lane [*n* = 3; mean±standard error of the mean (s.e.m.)]. (C) Silencing of the phosphatase PP2A by individual siRNAs. Asynchronously growing HeLa cells were transfected with either a pool of four individual siRNAs targeting human *PPP2CA* (si*PPP2CA* pool) or each of the siRNAs separately and processed for western blotting 48 hours after the transfection. Extracts were blotted for the proteins as indicated. The intensity of the PP2A-Cα and PP2A-A signals were quantified for each condition and corrected for the Actin loading control (*n* = 3; mean±s.e.m.). (D) MASTL counteracts the activity of PP2A in mitosis. HeLa cells transfected with indicated siRNAs were synchronised in mitosis by thymidine synchronisation and taxol treatment. Mitotic cells were treated with 10 µM of RO-3306 for 90 minutes to obtain cells in G1 phase. Extracts were processed for western blot analysis and blotted for indicated proteins. The intensity of the cyclin B1 and p-Serine signal was quantified for each individual lane and corrected for the Actin loading control (*n* = 3; mean±s.e.m.). Note that the APC3 phosphorylation is restored upon co-depletion of *MASTL* and *PPP2CA*.

To reveal the role of MASTL in phosphorylation events in mitosis, we silenced *MASTL* by RNAi and then collected a pure fraction of mitotic cells (Fig. 1A, left panel). First, we analysed the status of threonine phosphorylation of mitotic epitopes by western blots. Phospho-threonine epitopes were, on average, two-fold reduced in *MASTL*-depleted mitotic cells as compared to control mitotic cells (Fig. 1A, second panel; the table shows relative lane density is 0.55 in si*MASTL* cells compared to siCTRL). The depletion of *MASTL* reduced the overall spectrum of cyclin B1-Cdk1 substrates, as shown by the quantification of individual bands recognised by the phospho-threonine antibody (Fig. 1A, right table). *MASTL* depletion led to a similar reduction of total mitotic phospho-serine epitopes (Fig. 1B). Further quantification of the phospho-serine signal showed that while *MASTL* depletion reduced mitotic phosphorylations (61%±0.02% of siCTRL), phospho-serine levels in G2 phase were maintained, so these occurred mostly independently of MASTL [8%±0.02% (si*MASTL*) or 10%±0.03% (siCTRL)] (Fig. 1B, right graph).

To investigate whether the effects of *MASTL* silencing in human cells involved PP2A, we tested the effects of reducing the expression levels of the catalytic subunit of PP2A alpha (PP2A-Cα) (Fig. 1C). Silencing of *PPP2CA* by individual siRNAs showed a moderate depletion of PP2A-Cα at the protein level (Fig. 1C, western blot and left graph; a maximum of 25% reduction). This reduction in PP2A-Cα expression correlated with a significantly reduced expression of the PP2A scaffold subunit, PP2A-A (Fig. 1C, right graph). Next, we tested the combined depletion of *MASTL* and *PPP2CA* (Fig. 1D). *MASTL* silencing specifically reduced mitotic phosphorylations by 41% in taxol-arrested cells that were collected by mitotic shake-off. In line with PP2A being the predominant target of MASTL, co-depletion of *PPP2CA* restored the phosphorylation of APC3 but also the general phosphorylation pattern of cyclin B1-Cdk1 substrates, increasing from 59%±0.06% to 82%±0.03% (Fig. 1D, western blot shows supershifted phospho-APC3 and left graph shows total phosphorylation). Interestingly, *MASTL* RNAi cells exited mitosis before cyclin B1 was degraded (Fig. 1D). In G1 phase, roughly 28%±0.04% of the initial amount of cyclin B1 remained after forcing mitotic exit (90 minutes of RO-3306 treatment), as compared to approximately 6%±0.03% in siCTRL cells (Fig. 1D, right graph). Importantly, combined RNAi of *MASTL* and *PPP2CA* partially restored normal cyclin B1 degradation [12%±0.02% (si*PPP2CA* + si*MASTL*) compared to 6%±0.03% (siCTRL)]. Collectively, these data indicate that MASTL enforces cyclin B-Cdk1-mediated substrate phosphorylation in mitosis, by inhibiting the counteracting phosphatase activity of PP2A. This appears to be essential for efficient destruction of the APC/C^Cdc20^ substrate cyclin B1 upon mitotic exit.

### MASTL preferentially supports APC/C-mediated cyclin B1 destruction

**Fig. 2.**
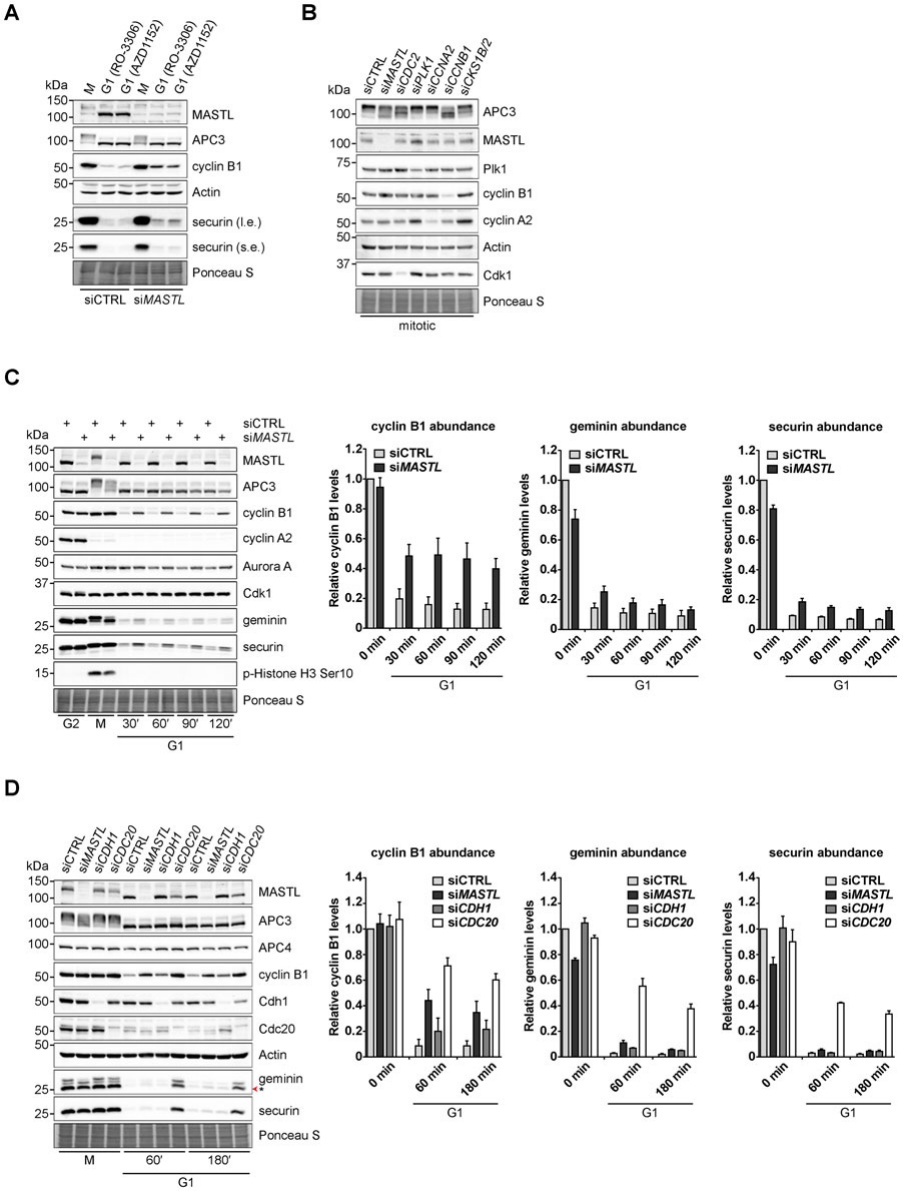
MASTL-mediated PP2A inhibition in mitosis is essential to direct cyclin B1 destruction by the APC/C. (A) Forced mitotic exit by either Aurora B or Cdk1 inhibition stabilises cyclin B1 in *MASTL* RNAi cells. HeLa cells were synchronised in mitosis by thymidine synchronisation and taxol treatment. Mitotic cells were treated with 10 µM of RO-3306 (Cdk1 inhibitor) or 1 µM of AZD1152 (Aurora B inhibitor) for 90 minutes to obtain cells in G1 phase. Extracts were processed for western blot analysis and blotted for indicated proteins. (B) APC/C phosphorylation in mitosis critically depends on cyclin B1-Cdk1 activity. HeLa cells transfected with indicated siRNA pools were synchronised in mitosis by a thymidine block followed by subsequent taxol treatment. Cells arrested in mitosis were collected by mitotic shake-off. Extracts were processed for western blot analysis either 24 hours (*PLK1* RNAi) or 48 hours after the siRNA transfection and blotted for the proteins as indicated. (C) Enforcing mitotic exit in absence of MASTL severely impairs the proteolysis of cyclin B1. HeLa cells transfected with indicated siRNAs were synchronised in mitosis by thymidine synchronisation and taxol treatment. Mitotic cells were treated with 10 µM of RO-3306 for indicated time points to obtain cells in G1 phase. Extracts were processed for western blot analysis and blotted for indicated proteins. The intensity of the cyclin B1, geminin, and securin signals were quantified for each condition and corrected for a background band, which was used as loading control (*n* = 3; mean±s.e.m.). (D) *MASTL* depletion selectively stabilises cyclin B1 in G1 phase, independent of general APC/C activity. HeLa cells transfected with indicated siRNAs were synchronised in M phase by thymidine synchronisation and taxol treatment. Mitotic cells were treated with 10 µM of RO-3306 for indicated time points to obtain cells in G1 phase. Extracts were processed for western blot analysis and blotted for indicated proteins. The asterisk (*) indicates the remaining securin signal after reprobing the blot with anti-geminin. The intensity of the cyclin B1, securin, and geminin signals were quantified for each condition and corrected for the Actin loading control (*n* = 3; mean±s.e.m.).

To further determine whether the destruction of APC/C substrates related to impaired APC/C phosphorylation, HeLa cells were monitored by western blot analysis of their extracts taken at different time points after inducing exit from mitosis. Therefore, a pure fraction of mitotic cells, arrested in taxol, was collected by mitotic shake-off and analysed, or treated with a Cdk1 inhibitor (RO-3306) or an Aurora B inhibitor (AZD1152), which induce spindle checkpoint override and force mitotic exit (Fig. 2A). *MASTL* RNAi prevented the efficient destruction of the APC/C^Cdc20^ substrate cyclin B1 upon mitotic exit, while securin destruction was less affected. Suppression of *MASTL*, *CDC2*, *CCNB1*, or a combination of *CKS1B* and *CKS2*, impaired APC/C phosphorylation in mitosis, detected by the incomplete phospho-shift of APC3 on western blot in fractions of purified mitotic cells (Fig. 2B). Cyclin B1-Cdk1, in a manner dependent on the Cdk1 subunit Cks, phosphorylates the APC/C subunit APC3 in mitosis (Kraft et al., 2003; Patra and Dunphy, 1998). Interestingly, in *MASTL*-depleted cells, cyclin B1 did not disappear for at least two hours after mitotic exit [Fig. 2C; western blot and left graph; 40%±0.07% cyclin B1 remained 120 minutes after mitotic exit (si*MASTL*) compared to 13%±0.04% (siCTRL)]. Securin and geminin levels followed a similar trend, but were clearly not stabilised as efficiently as cyclin B1 (Fig. 2C; securin levels after 120 minutes: 13%±0.02% in si*MASTL* compared to 7%±0.01% in siCTRL; geminin levels after 120 minutes: 13%±0.02% in si*MASTL* compared to 9%±0.04% in siCTRL). We conclude that either the APC/C is not properly activated by Cdc20 when *MASTL*-depleted cells enter mitosis, or the destruction of cyclin B1 is more dependent on MASTL than that of other Cdc20 substrates.

In order to investigate whether general APC/C activity was compromised due to a decrease in APC/C phosphorylation, we then compared the effects of *CDC20*, *CDH1*, and *MASTL* RNAi on APC/C substrate degradation (Fig. 2D). Suppression of *CDC20* expression resulted in stabilisation of cyclin B1 in G1 phase, after forcing mitotic exit of taxol-arrested cells [180 minutes G1: 60%±0.05% (si*CDC20*), respectively, compared to 9%±0.04% (siCTRL)]. *MASTL* depletion stabilised cyclin B1 protein levels to 35%±0.09%, up to at least 180 minutes after mitotic exit. Importantly, the degradation of securin and geminin was strongly sensitive to *CDC20* depletion, whereas RNAi of *MASTL* hardly influenced their protein disappearance after mitotic exit [securin or geminin levels at 180 minutes G1: 2.2%±0.01% or 2.0%±0.01% (siCTRL); 4.4%±0.01% or 5.7%±0.01% (si*MASTL*); 4.3%±0.01% or 4.9%±0.01% (si*CDH1*); 34%±0.02% or 38%±0.04% (si*CDC20*)]. These data confirm that general APC/C^Cdc20^ inhibition differs from APC/C^Cdc20^ inhibition as a consequence of *MASTL* depletion. While MASTL slightly supports APC/C^Cdc20^ activity during mitotic exit, it appears to play a more prominent role in the promotion of cyclin B1 destruction.

### APC/C-Cdc20 recruits cyclin B1 in prometaphase

**Fig. 3.**
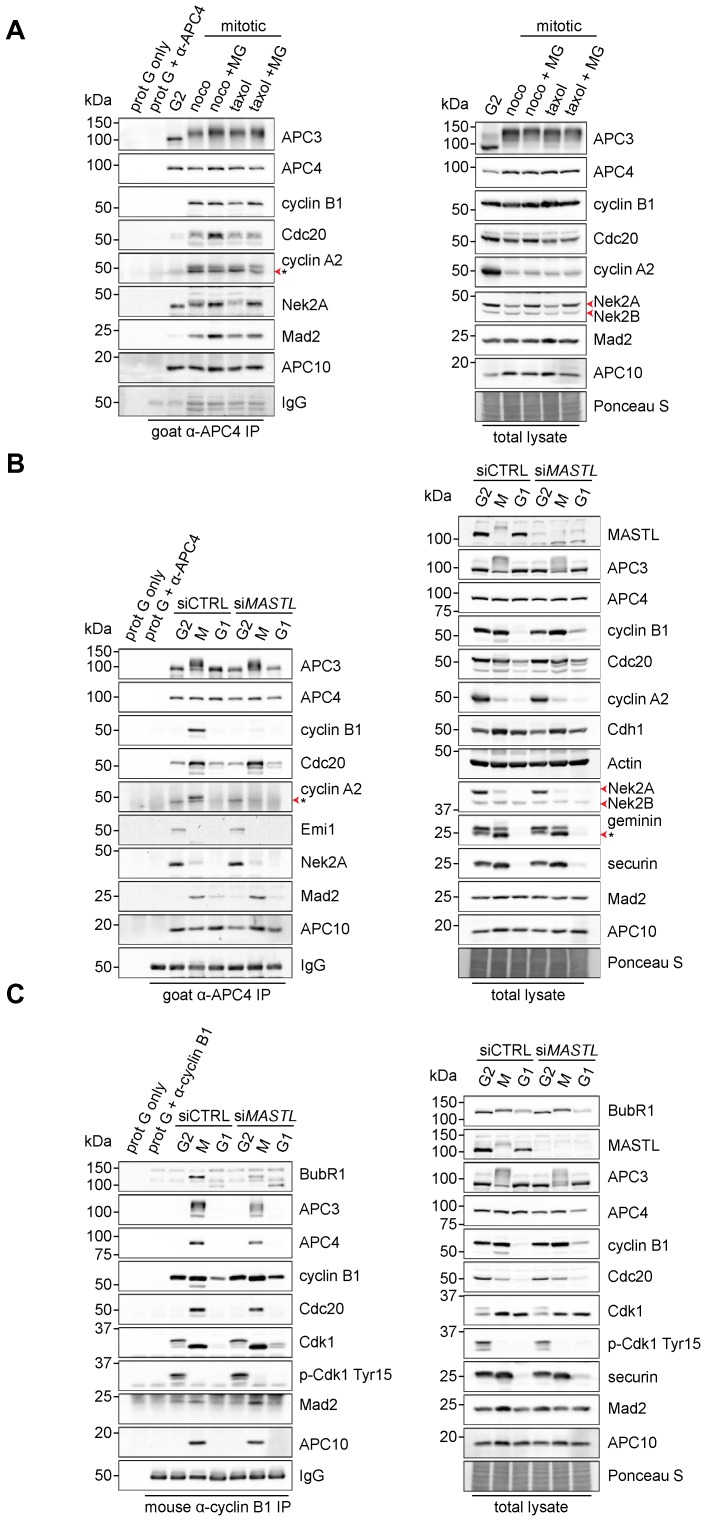
*MASTL* depletion significantly impairs cyclin B1 recruitment to the mitotic APC/C. (A) Cyclin B1 binds to the APC/C specifically in mitosis. HeLa cells were synchronised in G2 and M phase by a thymidine block, followed by nocodazole or taxol treatment. Cells arrested in mitosis were collected by mitotic shake-off, and treated for another 2 hours with 5 µM MG132 where indicated. Cell extracts were subjected to immunoprecipitation using a goat anti-APC4. Aliquots of the immunoprecipitates were analysed by western blotting. (B) *MASTL* silencing impairs the recruitment of cyclin A2 and cyclin B1 to the mitotic APC/C. HeLa cells transfected with indicated siRNAs were synchronised in G2 phase and mitosis using thymidine and taxol. Cells arrested in mitosis were obtained by mitotic shake-off and lysed directly or subsequently treated with 10 µM of RO-3306 for 90 minutes to obtain G1 phase cells. Cell extracts were subjected to immunoprecipitation using a goat anti-APC4 and aliquots of the immunoprecipitates were analysed by western blotting. Note that equal amounts of APC4 are immunoprecipitated in the mitotic fractions, whereas cyclins A2 and B1 bound to the APC/C are hardly detectable in the *MASTL* RNAi extracts. The asterisk (*) indicates the remaining securin signal after reprobing the blot with anti-geminin (right-hand panel). (C) Direct binding of cyclin B1 to the APC/C may depend on extensive APC3 phosphorylation in mitosis. HeLa cells treated as in (B), were subjected to immunoprecipitation using a mouse anti-cyclin B1. G1 phase cells were harvested 90 minutes after treatment with 10 µM of RO-3306. Aliquots of the immunoprecipitates were analysed by western blotting. For cyclin A2 blots in A,B, * denotes an aspecific band that is only observed upon immunoprecipitation.

We then reasoned that stabilisation of cyclin B1 may depend on the recruitment of cyclin B1 by the mitotic APC/C. Previously, we reported that cyclin B1, bound to Cdk1 and Cks, is retained at the APC/C in prometaphase, in a manner strictly dependent on APC3 (van Zon et al., 2010). Indeed, when we performed immunoprecipitation experiments using an APC4 antibody, to pull-down the APC/C from mitotic HeLa cell extracts and analysed interacting proteins on western blots, we found that cyclin B1, like cyclin A2, specifically bound to APC/C^Cdc20^ in prometaphase (Fig. 3A). The binding of cyclins A2 and B1 to the APC/C was specific for mitosis, and occurred regardless of the strength of the spindle checkpoint (Fig. 3A; compare nocodazole- with taxol-arrested cells) (Collin et al., 2013). In strong contrast, the APC/C^Cdc20^ substrate Nek2A bound the APC/C already in G2 phase, in line with previous findings and our own recent results (Hames et al., 2001; Boekhout and Wolthuis, 2015) (Fig. 3A). Interestingly, the amounts of APC/C-bound cyclin B1 or Nek2A did not correlate with the amounts of Cdc20 bound to the APC/C, indicating that Cdc20 may not be required for their binding (Fig. 3A; compare fourth and fifth lanes).

We then tested whether MASTL is critical to direct cyclin B1 to the phosphorylated APC3 in prometaphase. We performed APC4 immunoprecipitations on extracts of cells synchronised in mitosis with an active spindle checkpoint after knockdown of *MASTL* (Fig. 3B). Cyclin B1 bound specifically to the APC/C immunoprecipitated from mitotic cells, but, strikingly, we hardly detected cyclin B1 at the mitotic APC/C after *MASTL* depletion. Identical observations were made with an APC4 antibody that has been described before in the investigation of APC/C binding proteins (Izawa and Pines, 2011) (supplementary material Fig. S1A). Importantly, this reduced affinity for the APC/C was also observed for cyclin A2, but not for Nek2A, Cdc20 or Mad2. In agreement with this, we found that the half-life of cyclin A2 in mitosis was increased upon *MASTL* silencing (supplementary material Fig. S1B). After prolonged mitosis (>4 hours), however, cyclin A2 eventually disappeared (Fig. 3B, right panel). In both control and *MASTL* RNAi cells we observed an unaltered stoichiometry of the APC/C, with comparable amounts of Cdc20 present. Moreover, we did not detect any Emi1, the interphase inhibitor of APC/C^Cdh1^ (Di Fiore and Pines, 2007; Machida and Dutta, 2007), in mitotic APC/C complexes (Fig. 3B, left panel). We conclude from these experiments that mitotic cyclins are processed more effectively when they are targeted to the phosphorylated APC/C, which is stimulated by MASTL. Such targeting depends on the small Cdk subunit Cks, which has a high affinity for the phosphorylated APC/C (Fig. 2B, Wolthuis et al., 2008; van Zon et al., 2010).

To further corroborate these findings, we performed immunoprecipitations of cyclin B1. This revealed APC/C binding in mitosis, which was reduced in *MASTL* RNAi cells (Fig. 3C). We observed reduced binding of cyclin B1 to APC3, APC4, APC10, or Cdc20 upon *MASTL* suppression. In all experiments shown here, we detected hypo-phosphorylation of APC3 in the *MASTL* RNAi extracts. Because we showed earlier that APC3 is essential for cyclin B1 binding to the APC/C, we conclude from these data that cyclin B1 in particular requires hyper-phosphorylation of APC3 to bind the mitotic APC/C.

**Fig. 4.**
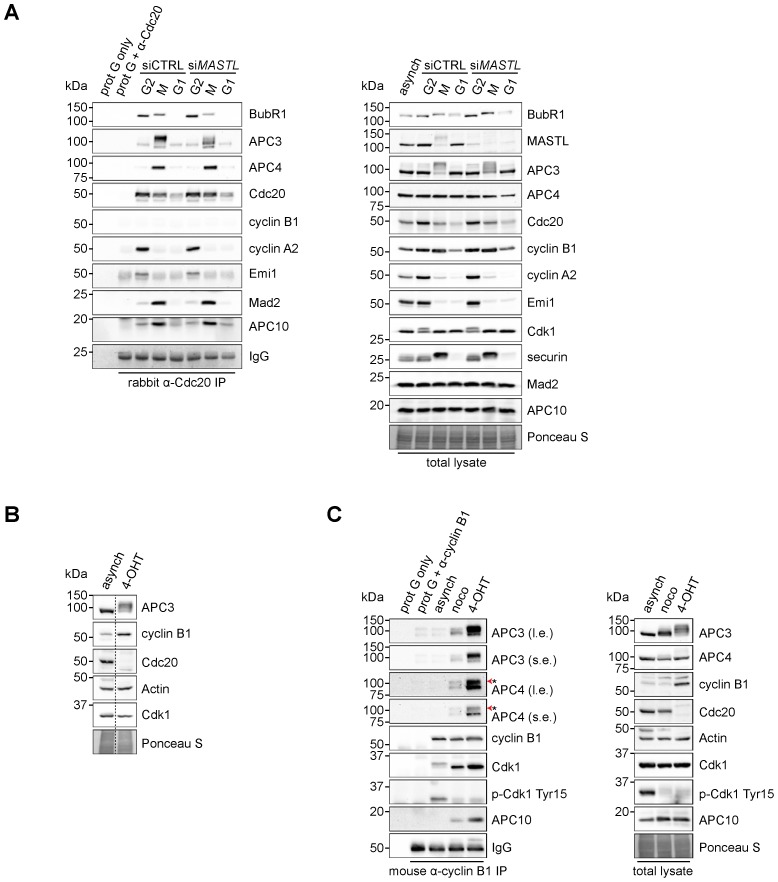
Cyclin B1 associates with the mitotic APC/C, independent of Cdc20. (A) Cyclin B1 recruitment to the APC/C does not involve direct Cdc20 binding, different from cyclin A2. HeLa cells transfected with indicated siRNAs were synchronised in G2 phase and mitosis using thymidine and taxol. Cells arrested in mitosis were obtained by mitotic shake-off and lysed directly or subsequently treated with 10 µM of RO-3306 for 90 minutes to obtain G1 phase cells. Cell extracts were subjected to immunoprecipitation using a rabbit anti-Cdc20 and aliquots of the immunoprecipitates were analysed by western blotting. Asynch refers to an asynchronous, non-transfected lysate. (B) Genetic ablation of *CDC20* results in a mitotic arrest due to cyclin B1 stabilisation. Cdc20(Δ/Δ); RERT(+/Cre) mouse embryonic fibroblasts (MEFs) were asynchronously grown or thymidine synchronised for 9 hours and treated with 1 µM of 4-hydroxytamoxifen (4-OHT) to induce Cre activity, resulting in the excision of exon 2 of *CDC20* (Cdc20(Δ) allele). Cells arrested in mitosis by the 4-OHT treatment were obtained by mitotic shake-off. Extracts of asynchronous or 4-OHT treated cells were subjected to western blot analysis. The dashed line indicates where two lanes have been placed next to each other. (C) Cyclin B1 binds the mitotic APC/C regardless of the presence of Cdc20. Cdc20(Δ/Δ); RERT(+/Cre) MEFs were either asynchronously grown or synchronised in mitosis using thymidine and nocodazole or 4-OHT treatment. Enrichment of cells arrested in mitosis was obtained by mitotic shake-off. MEF lysates were subjected to immunoprecipitation using a mouse anti-cyclin B1. Aliquots of the immunoprecipitates were analysed by western blotting. The asterisk (*) indicates the remaining APC3 signal after reprobing the blot with anti-APC4.

Does the binding of cyclin B1 to the mitotic APC/C depend on Cdc20? Previously, in *CDC20* RNAi experiments, we found that *CDC20* depletion by RNAi did not affect binding (van Zon et al., 2010). In line with that observation, the cyclin B1 D-box, which is known to bind Cdc20 with low affinity, was also not required. These results were questioned, however, in another study (Izawa and Pines, 2011). Here, we aimed to more thoroughly investigate this matter, and particularly to further address the role of MASTL and Cdc20 in directing cyclin B1 to the APC/C. Immunoprecipitation of Cdc20 specifically pulls down the APC/C in extracts from cells arrested in mitosis, but cyclin B1 is hardly detectable under these conditions (Fig. 4A). These immunoprecipitations were carried out under non-saturating conditions of the Cdc20 antibody, resulting in the isolation of mostly mitotic checkpoint complexes (MCCs) bound to the APC/C in mitosis. We showed before that only a fraction of the cellular pool of APC/C is found in complex with cyclin B1 (van Zon et al., 2010). We then used tamoxifen-inducible *CDC20* knockout cells (Manchado et al., 2010) to test the contribution of Cdc20 to the observed interaction between cyclin B1 and the APC/C more thoroughly. After genetic ablation of *CDC20*, these cells arrested in mitosis and could be collected by mitotic shake-off, for immunopreciptation experiments. Importantly, genetic ablation of *CDC20* did not prevent cyclin B1-APC/C complex formation in prometaphase, showing that Cdc20 is dispensable for cyclin B1 recruitment to the mitotic APC/C (Fig. 4B,C). Cyclin B1 can be recruited by phosphorylated APC/C, in a D-box–independent manner, and MASTL supports this. Recently, it was shown that this interaction between cyclin B and the mitotic APC/C plays a role in residual cyclin B1 degradation, observed after inhibiting the APC/C-Cdc20 interaction together with the Cdc20–D-box interaction, when combining two distinct APC/C inhibitors (Sackton et al., 2014).

### Anaphase bridges upon *MASTL* depletion may arise from incomplete separase activation

**Fig. 5.**
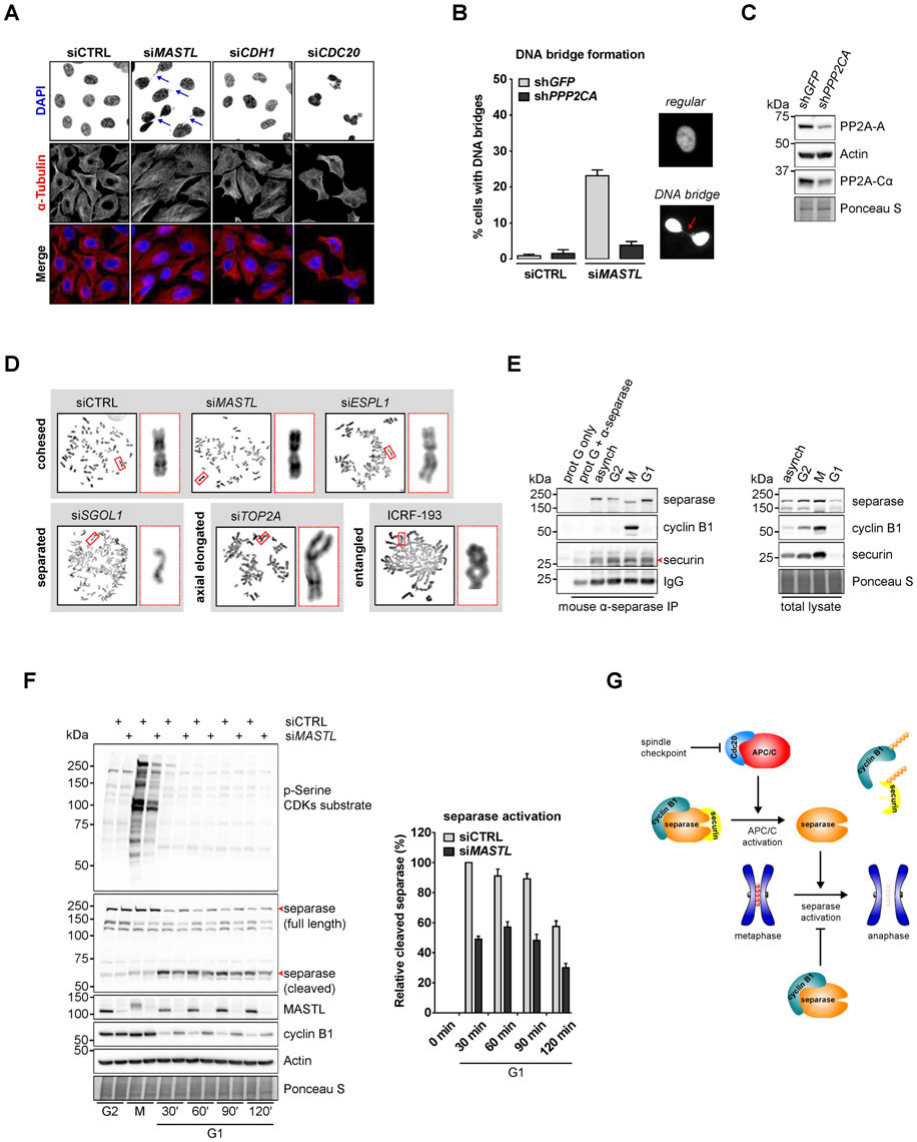
*MASTL* depletion increases the frequency of anaphase bridges that may depend on the activity of separase. (A) DNA bridges formed during anaphase are specific to *MASTL* silencing. Asynchronously growing HeLa cells were transfected with indicated siRNAs, formaldehyde fixed after 48 hours, and subjected to immunofluorescence staining using anti-α-Tubulin and DAPI. Arrows indicate DNA bridges connecting the daughter cells in G1 phase. (B) Combined depletion of *MASTL* and *PPP2CA* prevents the formation of DNA bridges. HeLa cells stably infected with an shRNA targeting *PPP2CA* (sh*PPP2CA*) or a control shRNA (sh*GFP*) were transfected with indicated siRNAs. Cells were formaldehyde fixed after 48 hours, and subjected to immunofluorescence staining using DAPI. Representative images show a regular cell or two daughter cells that are connected to each other by a DNA bridge (indicated by the red arrow). The amount of cells containing a DNA bridge were scored and quantified for each condition [*n* = 3; mean±standard deviation (s.d.)]. (C) Western blot analysis to show the effects of indicated shRNAs on total protein levels. A reduction in the protein levels of PP2A-A and PP2A-Cα demonstrates the knockdown efficiency of sh*PPP2CA*. (D) *MASTL* silencing does not alter the mitotic chromosome morphology. HeLa cells transfected with indicated siRNAs were synchronised in mitosis by a thymidine block followed by subsequent nocodazole treatment for 5 hours. Cells were processed for chromosome spreads 48 hours after transfection (or 24 hours in case of *SGOL1* RNAi). Where indicated, cells were treated for 13 hours with 5 µM ICRF-193. Chromosomes were stained using DAPI. The different treatments are categorised based on the mitotic chromosome morphology. Insets demonstrate the morphology of a representative chromosome for each condition. (E) Cyclin B1 and separase physically interact during mitosis. HeLa cells were synchronised in G2 phase and mitosis using thymidine and taxol. Cells arrested in mitosis were obtained by mitotic shake-off and lysed directly or subsequently treated with 10 µM of RO-3306 for 2 hours to obtain G1 phase cells. Cell extracts were subjected to immunoprecipitation using a mouse anti-separase and aliquots of the immunoprecipitates were analysed by western blotting. (F) Absence of MASTL impairs the activation of separase in mitosis. HeLa cells transfected with indicated siRNAs were synchronised in G2 and M phase by thymidine synchronisation and taxol treatment. Mitotic cells were treated with 10 µM of RO-3306 for indicated time points to obtain cells in G1 phase. Extracts were processed for western blot analysis and blotted for indicated proteins. The intensity of the separase cleavage product signal was quantified for each condition, starting in mitosis, and corrected for the signal of the same product present in mitotic lysates. The resulting amount was corrected for the total level of separase (full length plus cleavage product). Finally, these values were normalised to 100% at the onset (30 min G1 condition) of mitotic exit (*n* = 3; mean±s.e.m.). (G) Schematic working model of cyclin B1-mediated separase inhibition. The activation of APC/C^Cdc20^ results in the destruction of cyclin B1 and securin (indicated by the ubiquitin chains), thereby liberating separase from its inhibitors. Subsequently, activated separase triggers the induction of anaphase by cleaving centromeric cohesin (red dots). When cyclin B1 degradation is impaired, its binding to separase will prevent proper sister chromatid separation, leading to the DNA bridges observed in *MASTL* RNAi cells.

In a different line of investigation, when we compared the effects of either *CDC20* or *MASTL* RNAi by immunofluorescence, we had observed that only depletion of *MASTL* increased the incidence of DNA bridges, as detected by DAPI-positive threads connecting the G1 daughter cells (Fig. 5A). Partial *CDC20* depletion increased the amount of multinucleated cells, possibly due to a delay in metaphase, leading to premature sister chromatid separation as a consequence of cohesion fatigue (Daum et al., 2011; Stevens et al., 2011). However, neither si*CDC20*, nor si*CDH1* resulted in any detectable formation of DNA bridges (Fig. 5A; data not shown). These mitotic defects that arise upon *MASTL* RNAi are indicative of a premature exit from mitosis, most likely due to an increase in PP2A activity. We therefore tested whether co-depletion of *MASTL* and *PPP2CA* could restore the formation of DNA bridges between G1 daughter cells (Fig. 5B,C). Indeed, the effect of *MASTL* depletion was dependent on the presence of PP2A.

It has been shown previously that Gwl is needed for proper chromosome condensation in *Drosophila* (Yu et al., 2004). Defects in chromosome condensation may affect sister chromatid separation and subsequent cytokinesis (Steffensen et al., 2001). Others did, however, not observe clear chromosome condensation defects after *GWL* RNAi (Bettencourt-Dias et al., 2004). We therefore set out to investigate if MASTL could be important for DNA compaction in human cells, and whether this relates to the increased frequency of anaphase bridges upon its depletion (Voets and Wolthuis, 2010). To study this, we performed chromosome spreads of *MASTL* RNAi cells and compared the chromosomal compaction in these cells to that in cells depleted of other mitotic regulators of chromosomal topology, including human separase (*ESPL1*), Topoisomerase 2α (*TOP2A*), and shugoshin (*SGOL1*) (Fig. 5D). Suppression of *SGOL1* clearly induced premature sister chromatid separation of all chromosomes. Interestingly, however, cells entering mitosis after depletion of *MASTL* did not display any obvious alteration in chromosome morphology. In addition, chromosome cohesion seemed unaffected, as revealed by the highly similar chromosome appearance in control cells, or after *ESPL1* knockdown (Fig. 5D). Furthermore, depletion of *TOP2A* caused a clear elongation of the chromosome arms, different from catalytic inhibition of Topo2 using ICRF-193. Since ICRF-193 inhibits both Topo2α and Topo2β, this may explain the stronger effect on chromosome shortening that was observed after depletion of *TOP2A* alone. The knockdown efficiency of the different siRNAs was verified by western blotting (supplementary material Fig. S2). Taken together, these results indicate that depletion of *MASTL* has no major effect on mitotic chromosome compaction.

DNA bridges may also originate from the inability of the protease separase to cleave all cohesin rings that connect the centromeres (Buonomo et al., 2000; Chestukhin et al., 2003). The activity of separase is modulated on multiple levels. In mitosis, it is sequestered by securin, which safeguards against premature sister chromatid separation (Ciosk et al., 1998). In addition, however, both PP2A and cyclin B1 may regulate the activity of separase (Gorr et al., 2005; Holland and Taylor, 2006; Holland et al., 2007; Stemmann et al., 2001). In order to test whether cyclin B1 is able to interact with endogenous separase, we therefore performed separase immunoprecipitations on mitotic extracts (Fig. 5E). Securin bound to separase during all of the cell cycle stages tested. We even found residual binding to separase in G1 phase, when little securin was present, consistent with previous findings (Hellmuth et al., 2014). The binding of cyclin B1, however, was restricted to mitosis, indicating a mitosis-specific regulation of separase by cyclin B1. We also looked for endogenous PP2A binding, but did not detect an interaction with separase in immunoprecipitations.

Since depletion of *MASTL* results in the stabilisation of cyclin B1 after metaphase, we wondered whether this could influence separase activation. Therefore, we monitored separase activity in *MASTL* RNAi cells at different time points after the induction of mitotic exit (Fig. 5F). The activation of full-length separase can be traced by its autocatalytic processing, generating a cleaved fragment. The functional phenotype of the *MASTL* depletion is indicated by the reduced phospho-serine signal intensity. A quantification of the amount of cleaved separase (Fig. 5F; 49%±2.03% (si*MASTL*) of control cells after 30 minutes in G1), present at different times indicated that separase may not be not fully activated when MASTL is absent and could explain why these cells form anaphase bridges when passing through mitosis. We further investigated this model using a live-cell separase biosensor (Shindo et al., 2012), but found that the sensitivity of this approach was insufficient to detect the small differences in separase activity observed after *MASTL* depletion.

Taken together, we demonstrate that knockdown of *MASTL* strongly inhibits cyclin B1 degradation by perturbing the Cdc20-independent recruitment of cyclin B1 to the phosphorylated APC/C in prometaphase. Improper cyclin B1 degradation may have several consequences for late mitotic events, including a delay in separase activation, which may explain the increased incidence of anaphase bridges as a result of *MASTL* suppression (Fig. 5G).

## DISCUSSION

### Checkpoint-sensitive APC/C^Cdc20^ substrates require inhibition of PP2A for their efficient destruction

In agreement with our previous findings, we report here that MASTL, which acts though inhibition of PP2A, controls APC/C^Cdc20^ in mitosis. We found that hyper-phosphorylation of APC3 was primarily needed to efficiently degrade cyclin B1. We cannot rule out that our RNAi experiments disturb any biological processes prior to mitosis, but our data fit best to a model in which *MASTL* RNAi prevents effective phosphorylation of cyclin B1-Cdk1 substrates in mitosis. In mitotic cells, cyclin B1 binds APC/C^Cdc20^ in a D-box–dependent manner, which probably requires Cdc20 and APC10 (Izawa and Pines, 2011), but also in a D-box–independent manner, involving the phosphorylation of APC3, and binding of cyclin B1 to Cdk1 and Cks (van Zon et al., 2010). In contrast to previous findings (Chow et al., 2011; Izawa and Pines, 2011), we show that cyclin B1 binding to the prometaphase APC/C, as detected in immunoprecipitations, is independent of Cdc20. Genetic ablation of *CDC20* in Cdc20(Δ/Δ); RERT(+/Cre) MEFs completely abolished the expression of Cdc20. Under these conditions, we were able to efficiently pull down the APC/C complex together with cyclin B1. Hyper-phosphorylated APC3 mediates cyclin B1 recruitment to the APC/C while the spindle checkpoint is still active, which becomes effective after checkpoint release, when APC10 and Cdc20 direct cyclin B1 to the catalytic site of the APC/C (Carroll et al., 2005; Chang et al., 2014).

Our results reveal that the stability of geminin and securin, two other known APC/C^Cdc20^ substrates (Clijsters et al., 2013; Hagting et al., 2002), is less dependent on the phosphorylation state of the APC/C. Furthermore, compared to cyclin B1, securin destruction may be less dependent on APC3, as compared to APC2 (van Zon et al., 2010), consistent with the finding that the APC/C retains some activity in the complete absence of *APC3* (Thornton and Toczyski, 2003). However, depletion of *APC3* by RNAi does delay and inhibit the degradation of both cyclin B1 and securin (Izawa and Pines, 2011). We conclude that progressing through mitosis with PP2A prematurely activated inhibits APC/C^Cdc20^, but also interferes with recruitment of cyclin B1 to APC3. This results in less efficient destruction of securin, but impinges more drastically on cyclin B1 destruction.

### Anaphase DNA bridges: causes and consequences

Knockdown of *MASTL* results in severe mitotic defects that are likely the result of the decrease in cyclin B1-Cdk1 substrate phosphorylation. This is supported by the notion that knockdown of either *CCNA2* or *CCNB1* significantly impairs mitotic progression, and, eventually, also accurate mitotic exit during anaphase (Beamish et al., 2009; Chen et al., 2008; Kabeche and Compton, 2013). Moreover, straight Cdk1 inhibition in mitosis induces mitotic slippage, resulting in severe cytokinesis defects (Potapova et al., 2011; Vassilev et al., 2006). When cells exit mitosis in the absence of MASTL, even though cyclin B1 remains present, Cdk1 is thought to be rapidly inactivated by inhibitory phosphorylations at Thr^14^ and Tyr^15^ (Yu et al., 2006; Zhao et al., 2008). Despite the presence of cyclin B1 in anaphase, the spindle checkpoint is not re-activated in *MASTL*-depleted cells (Clijsters et al., 2014; Kamenz and Hauf, 2014; Rattani et al., 2014; Vázquez-Novelle et al., 2014). This is probably explained by the fact that cyclin B1-Cdk1 substrates are never properly phosphorylated in *MASTL* depleted cells, due to the incomplete repression of PP2A. Therefore, *MASTL* RNAi cells may inactivate Cdk1 sufficiently to exit mitosis without re-engagement of the spindle checkpoint even in case residual cyclin B1 remains.

Since Cdk1 phosphorylates many targets, it is difficult to dissect which one of these directs proper anaphase progression. It seems plausible that the combined action of multiple mitotic, Cdk1-regulated, factors fine-tune mitotic exit. We compared the effects of *MASTL* silencing to depletion of either *TOP2A* or *ESPL1*, which are both known to induce DNA bridges during anaphase (Carpenter and Porter, 2004; Chestukhin et al., 2003; Voets and Wolthuis, 2010). Suppression of either *MASTL* or *ESPL1* did not alter the chromosome morphology, which may relate to an insufficient depletion by RNAi. Indeed, genetic ablation of *MASTL* is likely to result in a more penetrant phenotype (Álvarez-Fernández et al., 2013). Knockdown of *TOP2A* resulted in elongated chromosome arms, consistent with previous observations (Farr et al., 2014). DNA compaction by Topo2α is enabled through the removal of entanglements as a result of its decatenation activity (Yanagida, 2009). So, the depletion of *TOP2A* increases the presence of DNA catenanes, resulting in the formation of DNA bridges during anaphase. Removal of *ESPL1* prevents the cleavage of centromeric cohesin, which holds the sister chromatids together in mitosis (Buonomo et al., 2000). This process is protected by shugoshin, and therefore elimination of *SGOL1* will result in loss of cohesion (Kitajima et al., 2004; Marston et al., 2004).

To maintain genomic stability, separase must be kept inactive before chromosome bi-orientation in metaphase. This is achieved by multiple mechanisms, including the binding to either securin or cyclin B1 (Ciosk et al., 1998; Gorr et al., 2005; Stemmann et al., 2001). In addition, PP2A has been shown to physically interact with separase, although this does not seem to regulate its catalytic activity (Holland et al., 2007). We propose that the excess of cyclin B1, present in *MASTL* RNAi cells, may prevent the normal activation of separase in metaphase. Additionally, securin is partially stabilised in these cells which may contribute to separase inhibition (Hellmuth et al., 2014). Accordingly, we observed that separase auto-cleavage is impaired once *MASTL* knockdown cells enter anaphase. An imbalance in separase activity may increase the frequency of anaphase bridges due to incomplete sister chromatid separation. As such, *MASTL* depletion may cause genomic instability.

### List of abbreviations

APC/C, anaphase-promoting complex/cyclosome; DIC, differential imaging contrast; Gwl, Greatwall kinase; MASTL, microtubule-associated serine threonine kinase-like; NEB, nuclear envelope breakdown; PP2A, protein phosphatase 2A.

## Supplementary Material

Supplementary Material
