# Supplementary material for: MASTL promotes cyclin B1 destruction by enforcing Cdc20-independent binding of cyclin B1 to the APC/C
Source: Biol Open. 2015 Mar 6;4(4):484–95. doi: 10.1242/bio.201410793 (PMC4400591; doi:10.1242/bio.201410793)
Supplement: Supplementary Material [file supp_4_4_484__index.html]

MASTL promotes cyclin B1 destruction by enforcing Cdc20-independent binding of cyclin B1 to the APC/C — MASTL promotes cyclin B1 destruction by enforcing Cdc20-independent binding of cyclin B1 to the APC/C — MASTL promotes cyclin B1 destruction by enforcing Cdc20-independent binding of cyclin B1 to the APC/C — Supplementary Material 

# MASTL promotes cyclin B1 destruction by enforcing Cdc20-independent binding of cyclin B1 to the APC/C

## bio.201410793 Supplementary Material

**Files in this Data Supplement:**

- Supplementary Material - Erik Voets and Rob Wolthuis doi: 10.1242/bio.201410793
