# Supplementary material for: MASTL promotes cyclin B1 destruction by enforcing Cdc20-independent binding of cyclin B1 to the APC/C
Source: Biol Open. 2015 Mar 6;4(4):484–95. doi: 10.1242/bio.201410793 (PMC4400591; doi:10.1242/bio.201410793)
Supplement: Supplementary Material [file supp_bio.201410793_bio.201410793-s1.pdf]

Biology Open (2015) 000, 1–12 doi:10.1242/bio.201410793

Supplementary Material

Erik Voets and Rob Wolthuis doi: 10.1242/bio.201410793

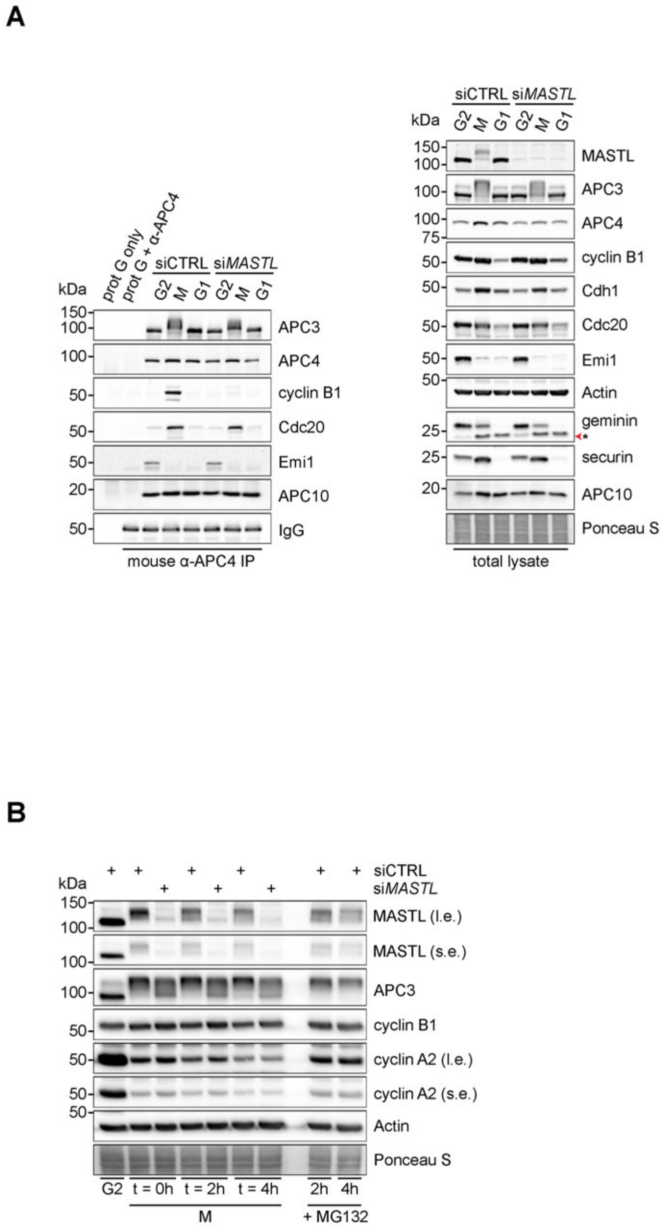

Fig. S1. Proper cyclin B1 recruitment to the mitotic APC/C requires the presence of MASTL. (A) *MASTL* silencing impairs the recruitment of cyclin B1 to the mitotic APC/C. HeLa cells transfected with indicated siRNAs were synchronised in G2 phase and mitosis using thymidine and taxol. Cells arrested in mitosis were obtained by mitotic shake-off and lysed directly or subsequently treated with 10 μM of RO-3306 for 2 hours to obtain G1 phase cells. Cell extracts were subjected to immunoprecipitation using a mouse anti-APC4 and aliquots of the immunoprecipitates were analysed by western blotting. The asterisk (*) indicates an aspecific background signal. (B) Cyclin A2 is partially stabilised upon *MASTL* depletion. HeLa cells transfected with indicated siRNAs were synchronised in G2 phase and mitosis using thymidine and nocodazole. Cells arrested in mitosis were obtained by mitotic shake-off and lysed directly or harvested at either 2 or 4 hours after the shake-off. Cells treated with 5 μM of MG132 for 2 or 4 hours serve as an internal control for the cyclin A2 stabilisation.

Biology Open

Biology Open (2015) 000, 1–12 doi:10.1242/bio.201410793

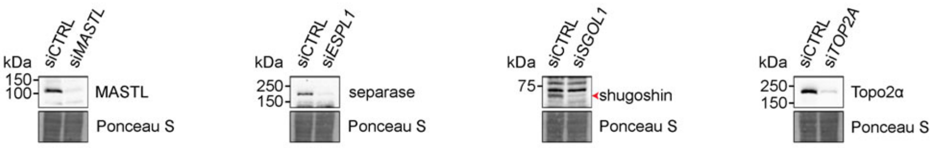

Fig. S2. Specificity of the siRNAs used for the chromosome spreads. Western blot analysis to show the effect of indicated siRNAs on total protein levels from HeLa cell lysates 24 hours (si*SGOL1*) or 48 hours after transfection.

Table S1. siRNA sequences used in this study

Gene name	Aliases	Accession_number	Oligo	Targeting sequence (5′-3′)
CCNA2	cyclin A2	NM_001237	J-003205-10	GGAAAUGGAGGUUAAAUGU
CCNA2	cyclin A2	NM_001237	J-003205-11	UAGCAGAGUUUGUGUACAU
CCNA2	cyclin A2	NM_001237	J-003205-12	AUGAGGAUAUUCACACAUA
CCNA2	cyclin A2	NM_001237	J-003205-13	UGAUAGAUGCUGACCCAUA
CCNB1	cyclin B1	NM_031966	J-003206-09	CAACAUUACCUGUCAUAUA
CCNB1	cyclin B1	NM_031966	J-003206-10	UGCACUAGUUCAAGAUUUA
CCNB1	cyclin B1	NM_031966	J-003206-11	GAAUGUAGUCAUGGUAAAU
CCNB1	cyclin B1	NM_031966	J-003206-12	CUAAUUGACUGGCUAGUAC
CDC2	Cdk1	NM_033379	J-003224-13	GGUUAUAUCUCAUCUUUGA
CDC2	Cdk1	NM_033379	J-003224-14	UCGGGAAAUUUCUCUAUUA
CDC2	Cdk1	NM_033379	J-003224-15	GUAUAAGGGUAGACACAAA
CDC2	Cdk1	NM_033379	J-003224-16	CAAACGAAUUUCUGGCAAA
CDC20	Cdc20/p55CDC	NM_001255	J-003225-14	CGGAAGACCUGCCGUUACA
CDC20	Cdc20/p55CDC	NM_001255	J-003225-15	GGGCCGAACUCCUGGCAAA
CDC20	Cdc20/p55CDC	NM_001255	J-003225-16	GAUCAAAGAGGGCAACUAC
CDC20	Cdc20/p55CDC	NM_001255	J-003225-17	CAGAACAGACUGAAAGUAC
CDH1	Cdh1	NM_016263	J-015377-06	CCACAGGAUUAACGAGAAU
CDH1	Cdh1	NM_016263	J-015377-07	GGAACACGCUGACAGGACA
CDH1	Cdh1	NM_016263	J-015377-08	GCAACGAUGUGUCUCCCUA
CDH1	Cdh1	NM_016263	J-015377-09	GAAGAAGGGUCUGUUCACG
CKS1B	Cks1	NM_001826	J-004586-06	CGACGAGGAGUUUGAGUAUUU
CKS1B	Cks1	NM_001826	J-004586-07	CAAAUUUACUAUUCGGACAUU
CKS1B	Cks1	NM_001826	J-004586-08	UCCAUUAUAUGAUCCAUGAUU
CKS1B	Cks1	NM_001826	J-004586-09	GGACAUAGCCAAGCUGGUCUU
CKS2	Cks2	NM_001827	J-007678-05	UCGACGAACACUACGAGUAUU
CKS2	Cks2	NM_001827	J-007678-06	CCAGAGAACUUUCCAAACAUU
CKS2	Cks2	NM_001827	J-007678-07	CCACAUAUUCUUCUCUUUAUU
CKS2	Cks2	NM_001827	J-007678-08	ACAAGCAGAUCUACUACUCUU
ESPL1	separase	NM_012291	J-004104-05	CCGAGGAUCACUUGAAAUA
ESPL1	separase	NM_012291	J-004104-06	GGAGAAGGCUCACAGUUAC
ESPL1	separase	NM_012291	J-004104-07	GAUCGUUUCCUAUACAGUA
ESPL1	separase	NM_012291	J-004104-08	GGAACGAAUUCUCUUUGUC
MASTL	Gwl/MASTL	NM_032844	J-004020-09	GGACAAGUGUUAUCGCUUA
MASTL	Gwl/MASTL	NM_032844	J-004020-10	ACUGGACGCUCUUGUGUAA
MASTL	Gwl/MASTL	NM_032844	J-004020-11	GCAAAUUGUAUGCAGUAAA
MASTL	Gwl/MASTL	NM_032844	J-004020-12	CCAUUGAGACGAAAGGUUU
PLK1	Plk1	NM_005030	J-003290-09	GCACAUACCGCCUGAGUCU
PLK1	Plk1	NM_005030	J-003290-10	CCACCAAGGUUUUCGAUUG
PLK1	Plk1	NM_005030	J-003290-11	GCUCUUCAAUGACUCAACA
PLK1	Plk1	NM_005030	J-003290-12	UCUCAAGGCCUCCUAAUAG
PPP2CA	PP2A-Cα	NM_002715	J-003598-09	CCGGAAUGUAGUAACGAUU
PPP2CA	PP2A-Cα	NM_002715	J-003598-10	ACAUUAACACCUCGUGAAU
PPP2CA	PP2A-Cα	NM_002715	J-003598-11	UCAUGGAACUUGACGAUAC
PPP2CA	PP2A-Cα	NM_002715	J-003598-12	CAGGUAGAGCUUAAACUAA
SGOL1	shugoshin/Sgo1	NM_001012413	J-015475-05	CAGCCAGCGUGAACUAUAA
SGOL1	shugoshin/Sgo1	NM_001012413	J-015475-06	GUUACUAUCUCACAUGUCA
SGOL1	shugoshin/Sgo1	NM_001012413	J-015475-07	AAACGCAGGUCUUUUAUAG
SGOL1	shugoshin/Sgo1	NM_001012413	J-015475-08	GUGAAGGAUUUACCGCAAA
TOP2A	Topo2α	NM_001067	J-004239-06	CGAAAGGAAUGGUUAACUA
TOP2A	Topo2α	NM_001067	J-004239-07	GAUGAACUCUGCAGGCUAA
TOP2A	Topo2α	NM_001067	J-004239-08	GGAGAAGAUUAUACAUGUA
TOP2A	Topo2α	NM_001067	J-004239-09	GGUAACUCCUUGAAAGUAA

siRNA targeting sequences used for transient knockdown were purchased from Thermo Fisher Scientific as set of four individual ON-TARGET-plus oligos.

Biology Open
